# Pose detection and localization of pineapple fruit picking based on improved litehrnet

**DOI:** 10.3389/fpls.2025.1577312

**Published:** 2025-05-21

**Authors:** Pinlan Chen, Bin Yan, Ganran Deng, Guojie Li, Zhende Cui, Shuang Zheng, Fengguang He, Ling Li, Xilin Wang, Sili Zhou, Shuangmei Qin, Zehua Liu, Ye Dai

**Affiliations:** ^1^ Agricultural Machinery Research Institute, Chinese Academy of Tropical Agricultural Sciences, Zhanjiang, Guangdong, China; ^2^ Key Laboratory of Tropical Agricultural Machinery, Ministry of Agriculture and Rural Affairs, Zhanjiang, Guangdong, China

**Keywords:** pineapple, keypoint detection, alphapose, litehrnet, lightweight, pick

## Abstract

To achieve accurate detection of the pineapple fruit picking area and pose under complex backgrounds and varying lighting conditions, this study proposes a pineapple keypoint detection model (LTHRNet) based on an improved LiteHRNet. Image data of pineapple fruits under different lighting conditions were collected, and six keypoints were defined to characterize the morphological features of the fruit. In the model design, LTHRNet incorporates the LKA_Stem module to enhance initial feature extraction, the D-Mixer module to capture both global and local feature relationships, and the MS-FFN module to achieve multi-scale feature fusion. In addition, the model employs parallel sub-networks with different resolutions to maintain high-resolution feature information and improve the precision and spatial accuracy of keypoint detection. Experimental results show that LTHRNet performs well in pineapple keypoint detection. It achieves 93.5% and 95.1% in KAP_0.5_ and KAR_0.5_, respectively, outperforming other models in terms of detection accuracy and robustness under challenging lighting and occlusion conditions, with a detection speed of 21.1 fps. For pose estimation, the average offset angle (AOA) of LTHRNet is 2.37°, which is significantly lower than that of other models. In summary, the proposed LTHRNet model demonstrates high accuracy and strong robustness in pineapple keypoint detection and pose estimation, providing reliable keypoint localization and pose estimation data for pineapple harvesting, while also offering an effective reference for pose recognition in other fruit-picking tasks.

## 1 Introduction

Pineapple is the third-largest tropical fruit in the world, with a global production of 29.96 million tons in 2023, according to Statista. In China, the planting area is approximately 1 million mu, and the production exceeds 2 million tons, accounting for about 7% of the world’s total pineapple planting area. Pineapple harvesting, a critical stage in its production chain, faces significant labor challenges. The supply of human resources for harvesting operations has become increasingly constrained as labor costs in rural areas continue to rise, and there is an outflow of young and middle-aged laborers (Li et al., 2010; He et al., 2024). Due to the physiological characteristics of pineapple fruit, the optimal harvesting period after fruit ripening typically lasts for one week, requiring laborers to work continuously during daylight hours, making the process highly labor-intensive. Consequently, the demand for intelligent pineapple harvesting equipment has become increasingly urgent. An intelligent pineapple harvesting system must integrate fruit information sensing, localization, robotic arm control, and path planning technologies (Chen et al., 2024), among which efficient fruit identification and localization are critical steps for enabling intelligent harvesting. However, the complex planting environment of pineapple, along with factors such as variations in light during the day, makes fruit pose detection difficult. Therefore, the development of an adaptable and accurate algorithm for pineapple fruit target detection and picking point localization is essential for advancing intelligent harvesting.

In recent years, both domestic and international researchers have conducted numerous studies on pineapple recognition. Traditional image processing methods typically rely on RGB image color space conversion, image segmentation, morphological processing, and other techniques (Chaivivatrakul and Dailey, 2014; Wu and Hua, 2016; Li, 2019). While these traditional algorithms are easy to implement, they are highly sensitive to environmental changes and exhibit poor robustness when handling complex images involving multiple fruits. With the advancement of computer technology and machine vision, deep learning has gradually been applied to the field of agricultural fruit harvesting, significantly improving the accuracy of fruit recognition (Chen et al., 2023; Song et al., 2023; Deng et al., 2025). Yu et al. (2020) proposed a strawberry pose detection algorithm using a rotating YOLO model, which introduces the rotational angle parameter of the target frame to determine the offset pose of the strawberry detection result. This model achieved a recognition accuracy of 94.43%. Liu et al. (2023) aiming to improve the accuracy of YOLOv3 for pineapple fruit recognition, integrated the Darknet-53 backbone network into the DenseNet of YOLOv3 and introduced the SPP-net in the feature output layer to enhance the information representation capability of the feature map. Li et al. (2023a) proposed an improved YOLOv7-tiny pineapple fruit detection model by incorporating CBAM in the backbone and neck network attention modules and replacing the CIoU loss function with SIoU, which increased model accuracy to 96.9%. However, in the complex pineapple planting environment, simply recognizing pineapple fruit and estimating the geometry of the picking point is insufficient to accurately determine the fruit’s picking pose.

Keypoint detection technology was initially applied to human pose recognition. Fang et al. (2022) proposed a top-down AlphaPose keypoint detection model, which can accurately recognize whole-body poses. This technology is now widely applied in agricultural production (Chen et al., 2024b; Zheng et al., 2024). Wu et al. (2023) proposed a top-down fruit stem localization method based on the growth characteristics of grapes. The positions of grape bunches were identified using YOLOv5, and keypoints of the grape bunches were further detected using HRNet combined with the Ghost module, achieving a final model recognition accuracy of 90.2%. Chen et al. (2024ac). introduced an improved YOLOv8n-Pose grape bunch keypoint detection model, which achieved a detection accuracy of 89.7%. Wang et al. (2024) used a keypoint detection algorithm combining YOLOv8n-Pose and LSKNet to identify and locate the picking points of wolfberry, achieving an average model accuracy of 92.7%. Jin et al. (2025) proposed CO-YOLO for pose detection of oil tea fruits, incorporating a MMA module to fuse multi-scale feature information and enhance perceptual capability, achieving a final precision of 90.6% and a recall of 87.0%. The keypoint detection model, which acquires the fruit picking pose, is more robust than traditional picking point localization methods based solely on target detection.

To address the challenge of pineapple fruit pose detection in complex background environments and varying lighting conditions, this study combines the YOLO target detection model (Khanam and Hussain, 2024) with the with the LiteHRNet keypoint detection model (Yu et al., 2021), proposing an improved LiteHRNet-based pineapple keypoint detection model. The keypoint data are then used to determine the 2D pose of the pineapple fruits and to detect the picking area, thereby providing technical support for the vision system of intelligent pineapple harvesting equipment.

## 2 Materials and methods

### 2.1 Image acquisition

**Figure 1:**
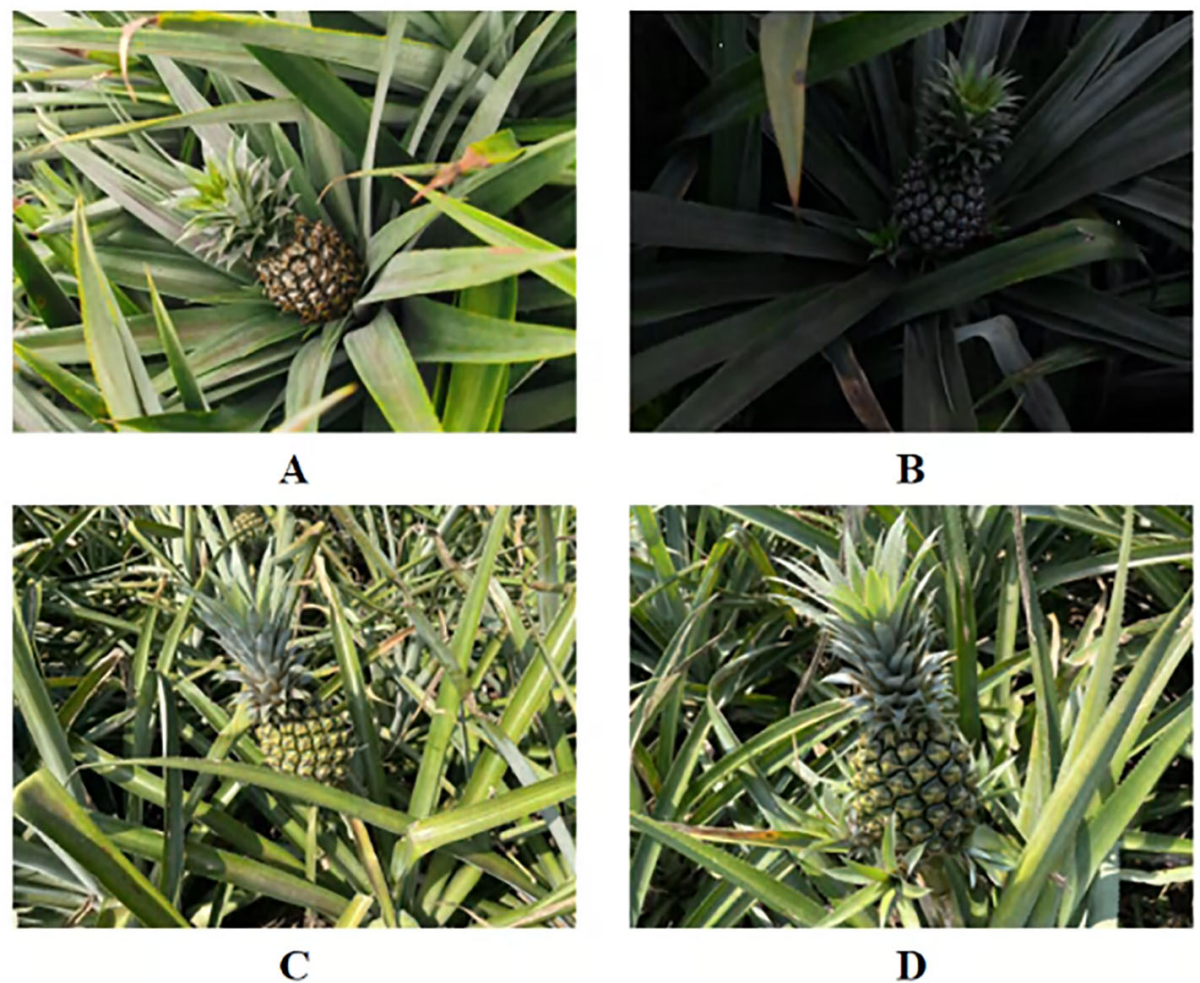
Example of pineapple dataset collection. **(A)** Cloudy. **(B)** Dusk. **(C)** Sunny. **(D)** Sunny with shadows.

The data for this study were collected in November 2024 from the pineapple land sea in Xuwen County(20°49′N, 110°30′E), Zhanjiang City, Guangdong Province, China. The distance between the camera and the pineapple fruit during image data acquisition ranged from 0.4 to 1 meter, with the camera angle tilted downward by approximately 45°. The camera used was a Hikvon industrial model, MV-CS060-10UC-PRO. During the image acquisition process, pineapple images were captured under different weather natural light conditions, including cloudy, dusk, sunny, and sunny with shadows, as shown in **Figure 1**. The planting area of ‘Comte de Paris’ pineapple in Xuwen County accounts for more than 90% of the total pineapple cultivation, while the remaining area is primarily dedicated to Tainong-series varieties (Zheng, 2023). The pineapple varieties included in the image dataset used in this study are Tainong 17 and ‘Comte de Paris’.

### 2.2 Pineapple dataset production

**Figure 2:**
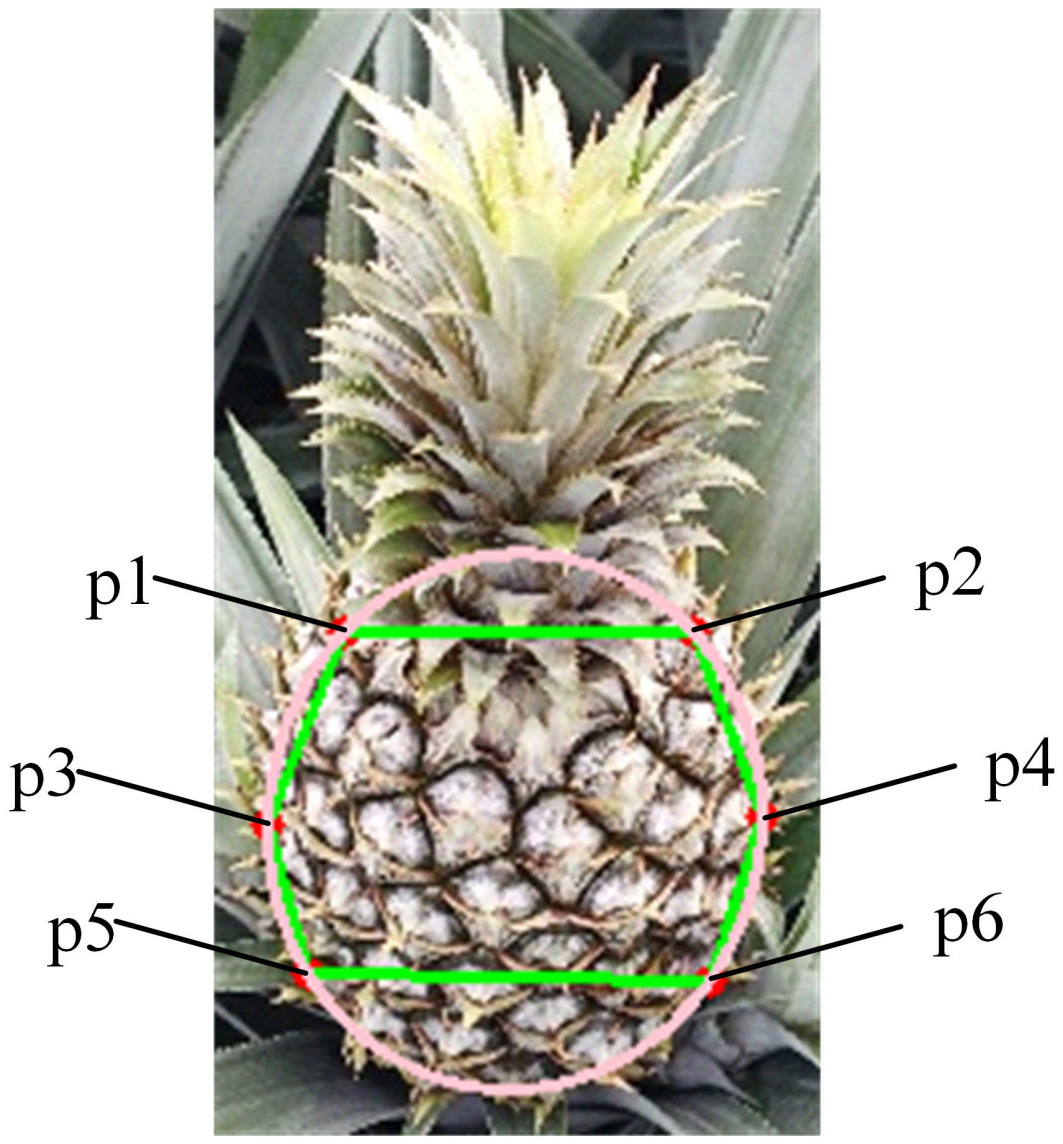
Pineapple keypoints definition. (p1–p6 represent the six keypoints that define the pineapple fruit shape).

The manual harvesting of pineapple fruits primarily involves grasping the fruit body and forcibly detaching it from the stem. Due to the influence of pineapple cultivation practices and environmental factors, the fruit body may exhibit tilting. Therefore, intelligent pineapple harvesting requires the vision system to accurately capture the fruit body region, and selecting appropriate keypoints to represent the morphological characteristics of the pineapple fruit body is a critical step in the visual localization system. In manual picking, workers primarily observe three key regions of the fruit body: the junction between the crown and the fruit body, the maximum width of the fruit (usually located in the middle), and the bottom of the fruit body. The pineapple fruit body generally exhibits an oval shape. Based on the areas of interest emphasized during manual picking, this study defines six keypoints that comprehensively represent both the picking region and the morphological structure of the pineapple fruit, as illustrated in **Figure 2**.

The defined keypoints, p1–p6, are located at the top-left, top-right, middle-left, middle-right, bottom-left, and bottom-right positions of the pineapple fruit contour, respectively. Specifically, p1 and p2 are situated at the parallel edges of the interface between the fruit body and the crown, reflecting the top of the grasping area. p3 and p4 are located at the middle of the fruit body, indicating the width of the pineapple’s midsection. p5 and p6 are positioned at the parallel edges of the interface between the fruit body and the stem, representing the bottom of the grasping area. An ellipse is fitted based on these keypoints, with the angle between the line connecting the midpoints of p1, p2 and p5, p6 and the Y-axis serving as the fruit’s offset. The irregular closed region formed by these keypoints is designated as the pineapple grasping region.

**Figure 3:**
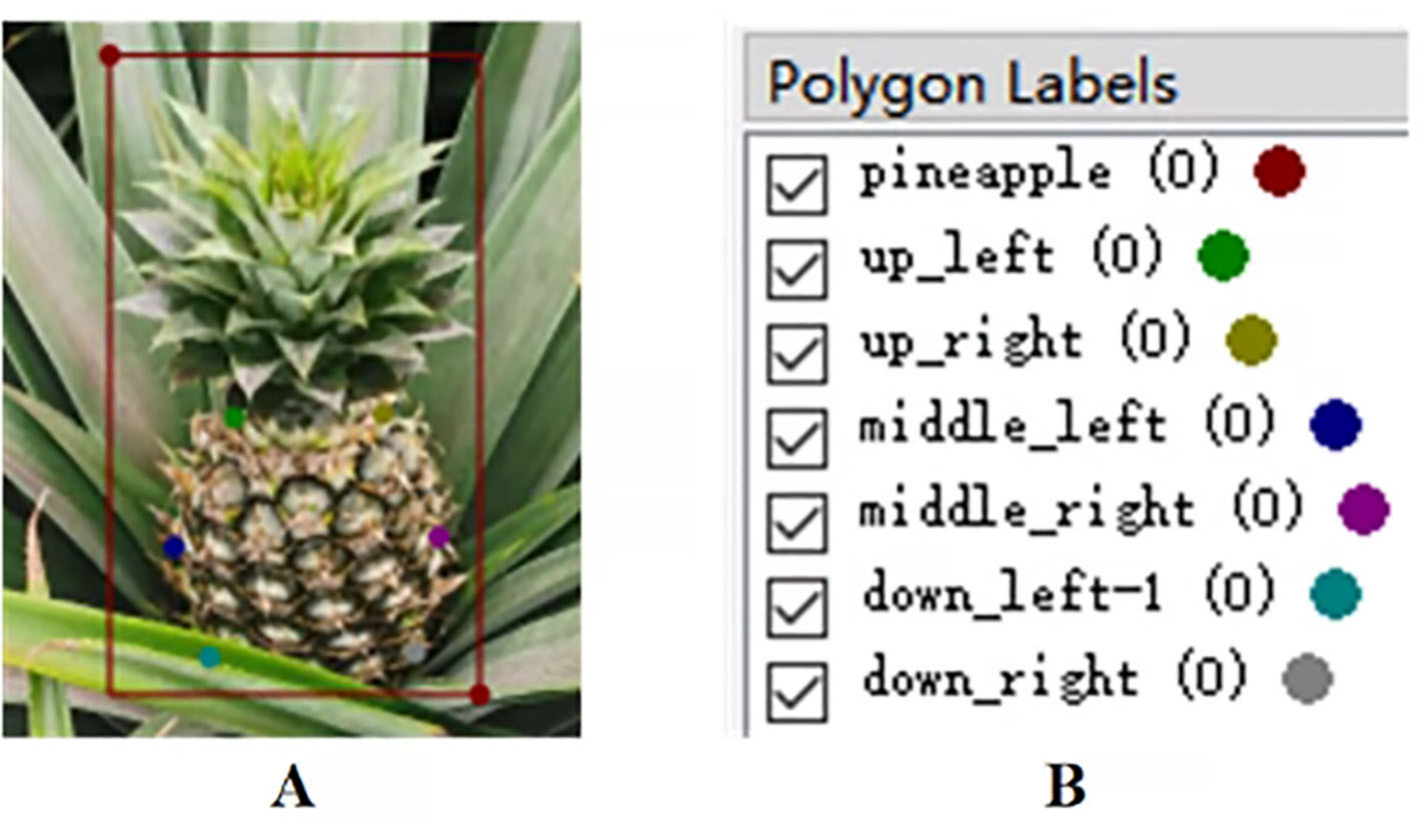
Pineapple image annotation. **(A)** Annotation. **(B)** Labels.

Based on the aforementioned definitions of keypoints, the Labelme annotation software was used to annotate the keypoints on the pineapples. The main body of the pineapple fruit and the entire crown(rectangle) were labeled as “pineapple.” The keypoints on the pineapple fruit, labeled as p1–p6, were named up_left, up_right, middle_left, middle_right, down_left, and down_right, respectively. For occluded keypoints, the suffix “-1” was appended to the label during annotation. When annotating multiple targets within the same image, the “group_id” was used for differentiation, as shown in **Figure 3**. Following this naming convention, a total of 3,964 images were annotated. Subsequently, the images were uniformly resized with a locked aspect ratio, adjusting the longer side to 640 pixels. Among them, 644 images were of the variety Tainong 17 and 3,320 images were of ‘Comte de Paris’. For model generalization performance testing, 150 images from each variety were selected, while the remaining 3,664 images were used as the main dataset. The JSON files obtained from the image annotations were then converted into the COCO dataset format. To ensure the stability of the model training process and enhance its generalization capability, the dataset was divided into training, validation, and test sets in an 8:1:1 ratio.

### 2.3 Pineapple keypoint detection methods

In fruit picking tasks, the vision system primarily employs three methods for fruit detection: object detection, semantic segmentation, and keypoint detection. Object detection provides only a bounding box, which often contains redundant regions and cannot precisely localize the picking area. Although semantic segmentation can classify pixels by category, it requires post-processing to extract the target point, which may introduce significant errors and becomes less reliable under fruit occlusion. In contrast, keypoint detection directly outputs the coordinates of keypoints within the picking area and can simultaneously determine the fruit pose. Therefore, for pineapple harvesting, keypoint detection provides a more robust solution for acquiring picking information.

Pose keypoint detection algorithms can generally be classified into two categories: top-down and bottom-up, with the top-down algorithm typically yielding higher detection accuracy. AlphaPose is a top-down keypoint detection model, and its detection accuracy exceeds that of models such as OpenPose (Qiao et al., 2017), YOLO (Khanam and Hussain, 2024), and others. To ensure the accuracy and reliability of the keypoint data extracted from the image, this study selects AlphaPose as the pineapple keypoint detection model for detecting the 2D keypoints of pineapple.

The detection accuracy of target detection plays a significant role in the overall accuracy of top-down algorithms, as pose estimation is performed within the target region. Consequently, mislocalization of the detection frame can lead to incorrect estimation of the target keypoints. At the core of AlphaPose, a Part-Guided Proposal Generator(PGPG) is introduced during the training process. This mechanism generates atomic poses by considering both the true frame and pose offset, thereby increasing the diversity of training samples for the Single Target Pose Estimator(STPE) in the backbone network. This allows the model to better detect incomplete target areas or keypoints, even in the case of occlusion, thus optimizing keypoint prediction accuracy. Furthermore, the feature heatmap generated by the target pose estimator contains redundant keypoints. To address this, AlphaPose introduces the parameterized Pose Non-maximum Suppression(PNMS), which enables the model to accurately eliminate redundant keypoints, even when the confidence threshold is low (Fang et al., 2022).

**Figure 4:**
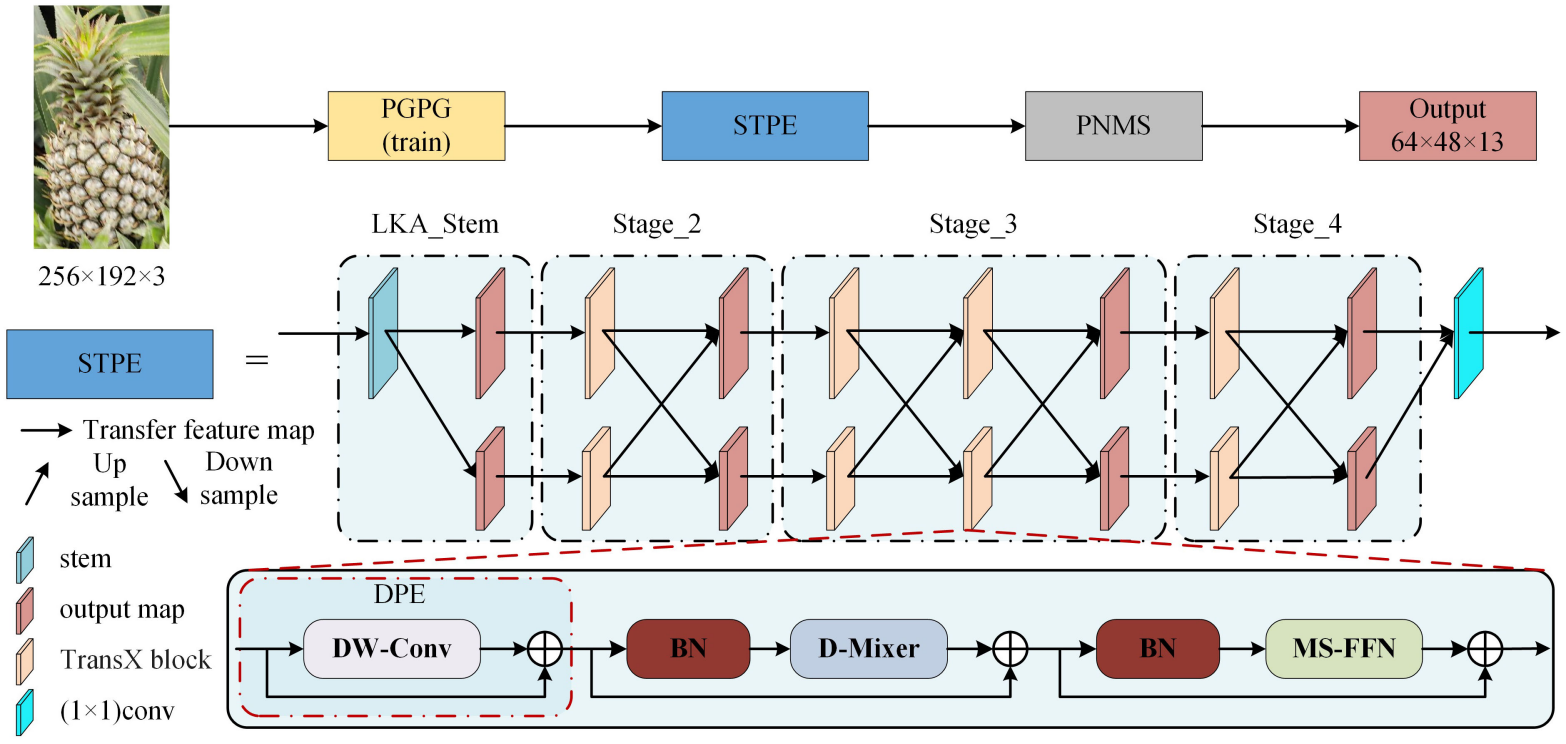
LTHRNet network structure. (The output map is the final output feature map of each section).

In comparison to the high-low-high resolution method used by traditional networks for feature extraction, LiteHRNet maintains high resolution throughout the process by connecting multiple subnetworks with different resolutions in parallel. This approach enhances both the accuracy and spatial precision of keypoint detection. Therefore, this study employs LiteHRNet as the STPE for AlphaPose. The Conditional Channel Weighting Block(CCWB) in LiteHRNet reduces the model’s parameters through the use of a shuffle block, but it also reduces the model’s characterization capability. To address this, the study replaces the CCWB with the TransX block (Lou et al., 2023) module, which consists of a Dynamic Position Encoding (DPE) (Li et al., 2023b), a Dual Dynamic Token Mixer(D-Mixer), and a Multi-scale Feed-forward Network (MS-FFN). Additionally, the LKA_Stem module is used to build the LiteHRNet input module, utilizing Large Kernel Attention(LKA) to further enhance the feature extraction performance. Furthermore, LiteHRNet is modified to reduce the CCWB module in stages 2–4 to prevent the loss of target feature information caused by excessive convolution. This reduction also decreases the number of network parameters, thereby improving model detection speed. The improved pineapple fruit keypoint detection network, Large Kernel TransX HRNet(LTHRNet), is shown in **Figure 4**. In this network structure, the 1, 2, and 1 TransX block modules are used for each branch of the network in stages 2-4. The resolution of each branch is 32×24 and 16×12, and the number of channels is set to 192 and 384, respectively.

#### 2.3.1 LKA_stem

**Figure 5:**
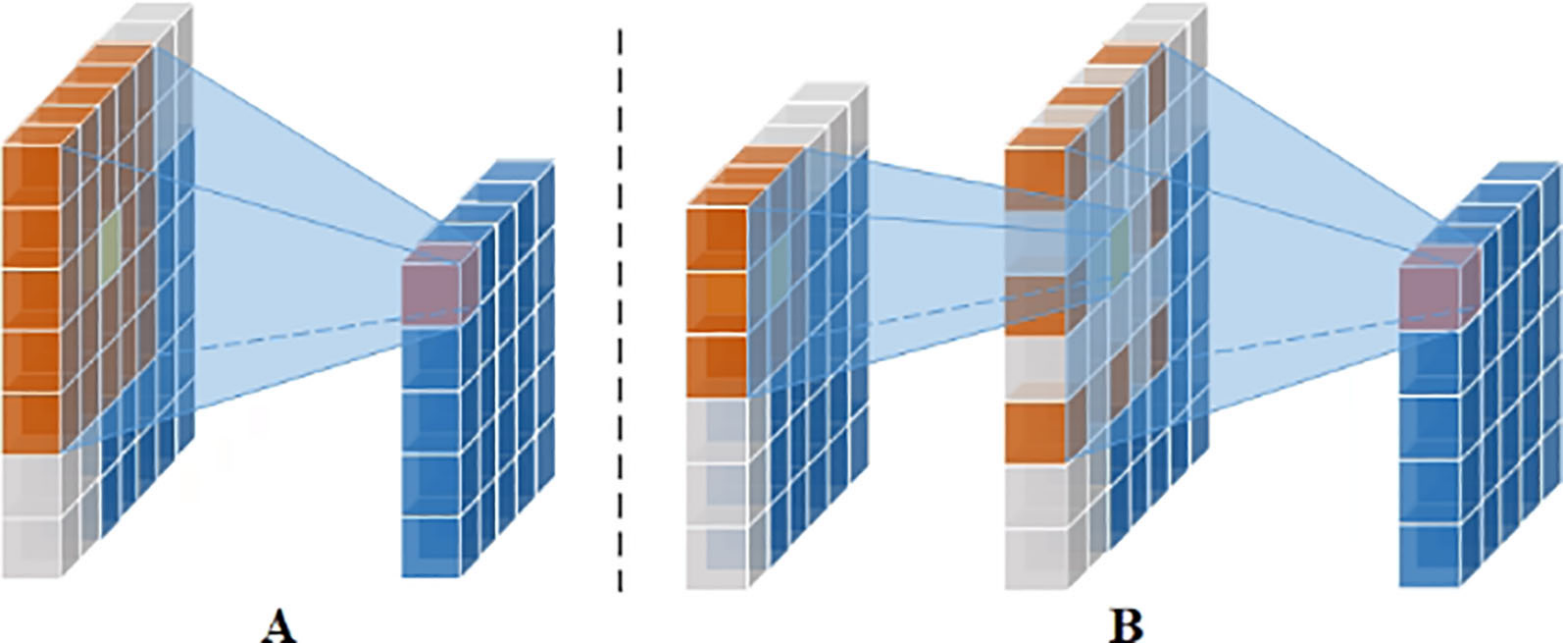
Single channel large kernel convolutional decomposition. **(A)** Standard conv. **(B)** Small kernel conv plus expansion conv. (Orange indicates convolution kernel size, yellow indicates the convolution kernel center, gray indicates the use of 0-fill, pink indicates features after convolution, and blue indicates features).

Attention mechanisms can selectively focus on or emphasize features that are more critical to the task, based on the dynamics of the input features, while suppressing or ignoring other parts. Large kernel convolution attention has a greater feature extraction capability, but it requires a significant amount of computation and parameters. To overcome this limitation, the large kernel convolution of a single-channel feature map can be decomposed into smaller kernel convolutions and expanded convolutions. As shown in **Figure 5**, for a 5×5 standard convolution of a single-channel feature map, it can be decomposed into a 3×3 convolution, a 3×3 inflated convolution with an expansion rate of 2, and further, a large kernel convolution of K×K can be decomposed into a small kernel convolution of (2d-1)×(2d-1), and an inflated convolution of $\left[\frac{K}{d}\right]$ × $\left[\frac{K}{d}\right]$ with an expansion rate of d, where [] represents the upward rounding function. This decomposition facilitates the connection of remote features and the estimation of the importance of points, all while maintaining low computational cost and parameter requirements.

According to depth-separable convolution, a standard convolution of a feature map with multiple channels can be decomposed into depth convolution and point convolution. LKA combines the single-channel large kernel convolution decomposition with depth-separable convolution to decompose the large kernel standard convolution of a multi-channel feature map into three components: spatially localized convolution(depth convolution, DW-Conv), spatially remote convolution(depth inflated convolution, DW-D-Conv), and channel convolution (point convolution, 1×1 Conv) (Guo et al., 2023). Thus, the LKA module can be represented as (Equation 1):


(1) $$Output=Conv_{1\times 1}\left(DW-D-Conv\left(DW-Conv\left(F\right)\right)\right)⊗F$$

Where F is the input feature map. When the convolution kernel is K, the expansion rate is d, and the number of channels is C, the number of module parameters P (Equation 2) is:


(2) $$P\left(K,d\right)={\left(2d-1\right)}^{2}\times C+{\left[\frac{K}{d}\right]}^{2}\times C^{2}+C^{2}$$

**Figure 6:**
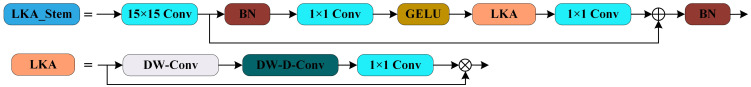
LKA_Stem module.

In this study, the LKA module with K=21 and d=3 is used, which corresponds to the decomposition into a 5×5 depth convolution and a 7×7 expansion with a 3-depth expansion convolution. Thus, the number of covariates is 25C + 50C², which is much smaller than the number of covariates for the standard convolution with K=21, 441C². The LKA_Stem (**Figure 6**) is passed through a conv layer with a step size of 8 for the 15×15 Conv, followed by batch normalization, 1×1 Conv, GELU activation function, LKA, another 1×1 Conv, and batch normalization for feature extraction.

#### 2.3.2 D-mixer

He keypoints defined are all located at the edge region of the pineapple fruit, where the similarity between the edge and the inner region is high. Therefore, the model needs to pay more attention to the global features of the input during the feature extraction process. The TransX block, designed based on Convolutional Neural Networks(CNN), is combined with Transformer to enhance the network’s expressive capability. The convolutional layer focuses on extracting local features, and its perceptual field is limited. By relying on multi-layer stacking, the global perceptual range is gradually expanded, but this may result in low efficiency when modeling long-range dependencies. On the other hand, Transformer models can directly capture relationships between pixels in an image through the self-attention mechanism, thus efficiently modeling long-range dependencies and global features. Both CNN and Transformer have distinct advantages in feature modeling, and their combination can complement each other to improve the overall performance of the model.

**Figure 7:**
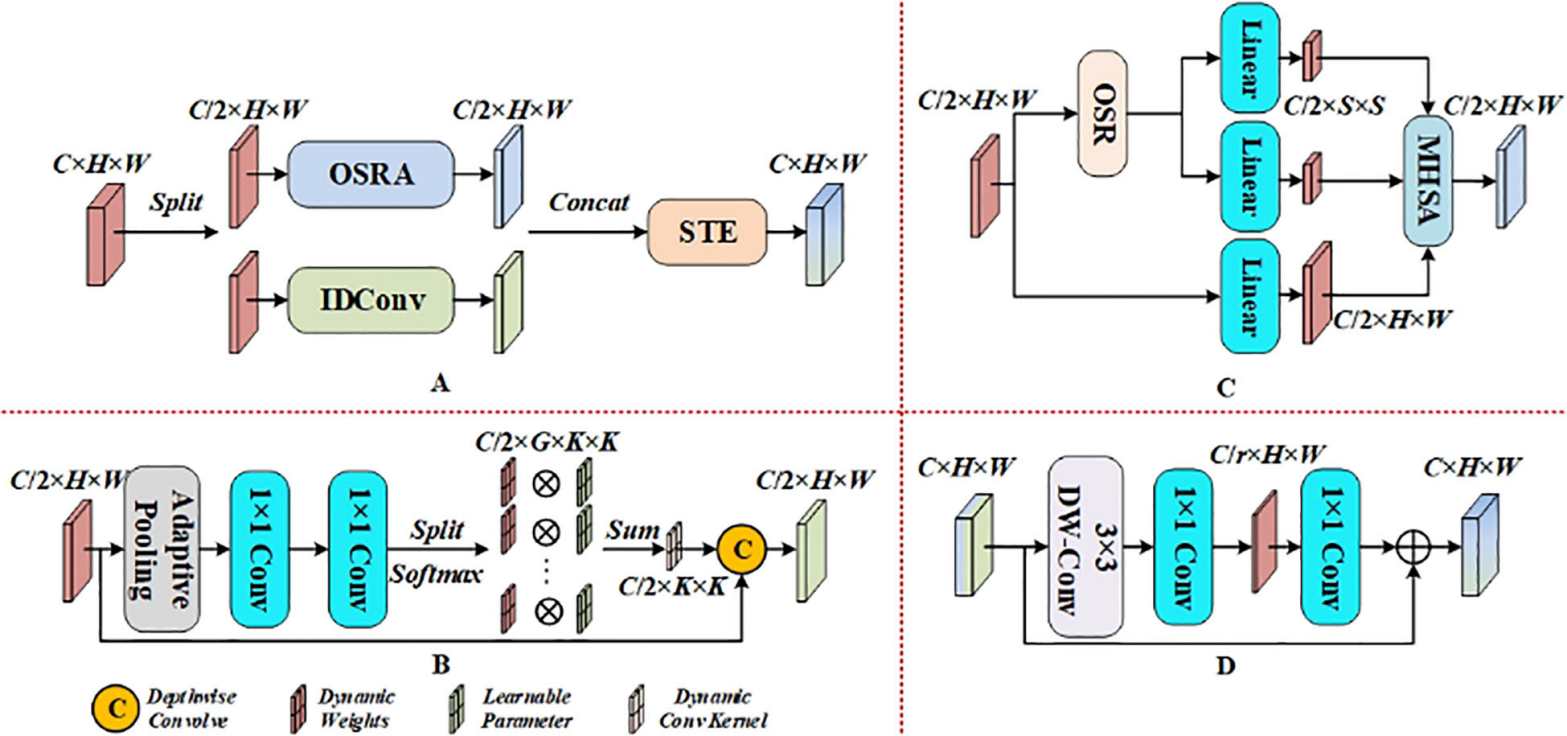
D-Mixer Module. **(A)** D-Mixer. **(B)** IDConv. **(C)** OSRA. **(D)** STE.


The D-Mixer module, through OSRA and IDConv, increases the effective sensory field of the network without reducing the long-range dependency of the input features. It dynamically utilizes both global and local information of the features and finally uses the Squeezed Token Enhancer(STE) for efficient feature fusion. The DMixer module is shown in **Figure 7** and is denoted as Equations 3, 4.

(3) $$F_{1},F_{2}=Split\left(F\right)$$

(4) $$Output=STE\left(Concat\left(OSRA\left(F_{1}\right),IDConv\left(F_{2}\right)\right)\right)$$

OSRA introduces depthwise separable convolution (Chollet, 2017) instantiated according to Overlapping Spatial Reduction(OSR) in Spatial Reduction Attention(SRA) (Wang et al., 2021). This approach effectively solves the problem of SRA’s global feature extraction in sparsely labeled regions, which otherwise results in non-overlapping space shrinkage. Such shrinkage destroys the spatial structure near the patch boundary, degrading the token quality. The method then uses multi-head self-attention(MHSA) to model remote dependencies. The weights of IDConv are dynamically generated based on the input features, enabling the convolution kernel to adjust adaptively according to the input data. This feature helps the model better adapt to complex, non-uniform feature distributions and is suitable for tasks that require the capture of both local and global information. Finally, STE employs deep convolution as well as channel compression and expansion to achieve cross-channel feature fusion at a low computational cost.

#### 2.3.3 MS-FFN

**Figure 8:**
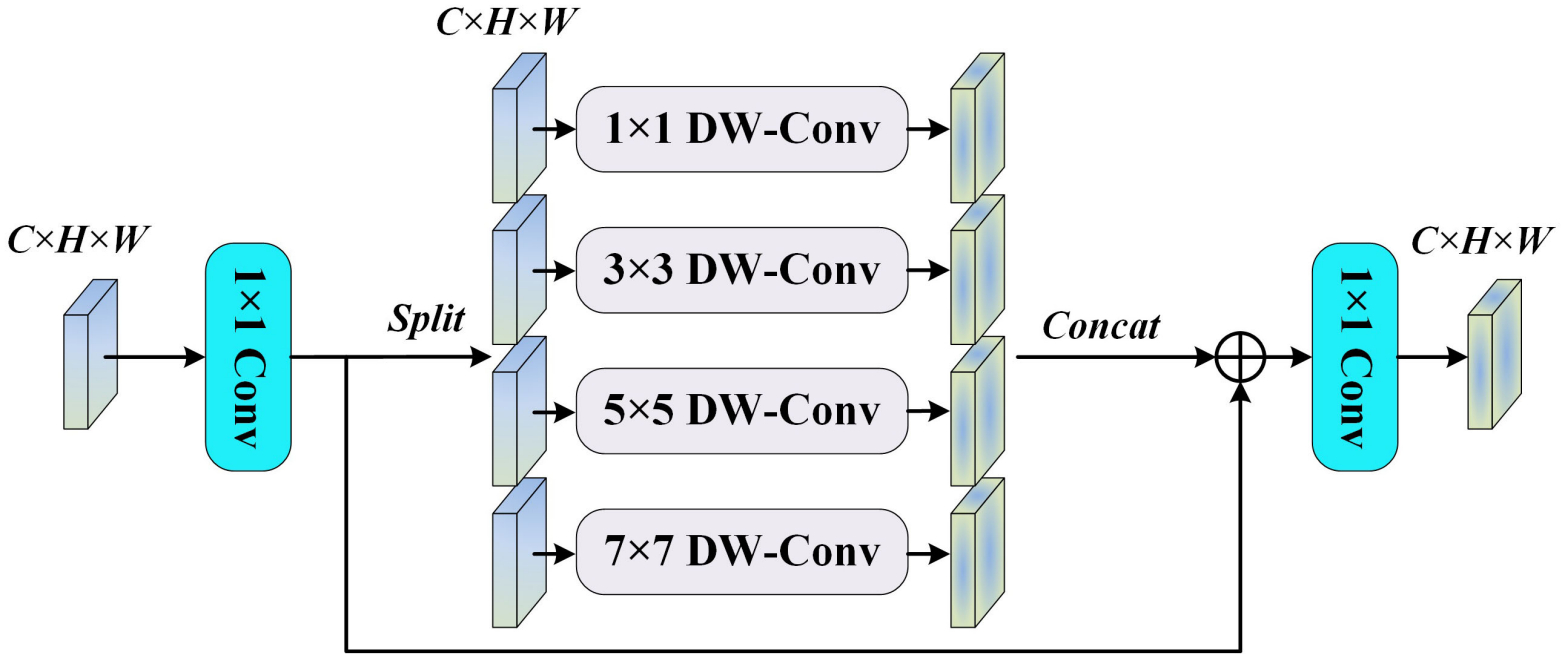
MS-FFN module.

Standard Feed-Forward Networks(FFN) typically employ single-scale linear transformations and activation functions to achieve feature transformation, which provides limited processing capability and struggles to capture cross-scale feature associations. Inverted Residual FFN reduces the number of parameters by using a bottleneck design and improves efficiency by combining with depth-separable convolution. However, its feature extraction capability depends on specific channel expansion and compression operations, which limits its ability to fuse cross-scale information. The Multi-Scale Feed-Forward Network (MS-FFN) places greater emphasis on extracting and fusing information at different scales. It is not limited to inter-channel transformation operations and is enhanced by multi-scale operations, which boost the model’s ability to adapt to complex features. MS-FFN is more effective at capturing the balance between global and local features.In this study, MS-FFN adopts a fourfold channel expansion along with deep convolution, utilizing convolution kernel sizes of 1, 3, 5, and 7. The 3×3, 5×5, and 7×7 deep convolutions effectively extract multi-scale features, while the 1×1 deep convolution optimizes and adapts the input features by adjusting channel information, as shown in **Figure 8**.

### 2.4 System processes

**Figure 9:**
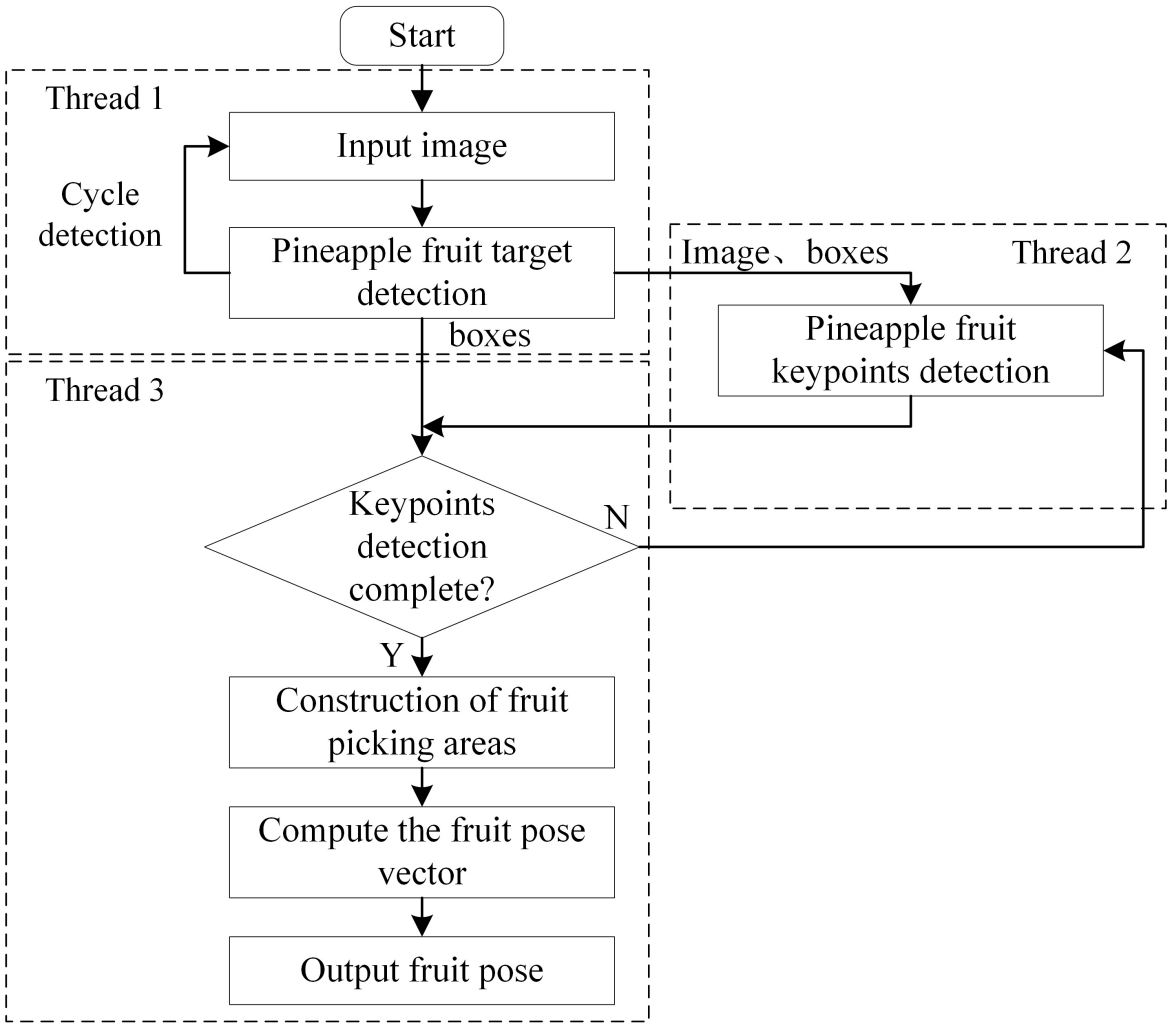
Flowchart of the pineapple fruit pose prediction system.

This study detects pineapple fruit pose information based on keypoint features, and the main system flow is shown in **Figure 9**. The system uses three threads running in parallel to accelerate processing, with data transferred and read between threads via queuing. First, thread 1 acquires the image input and applies the YOLO target detection algorithm to each frame, identifying all pineapple fruit target recognition boxes (bounding boxes) within the image. Thread 1 then sends the image with the detected target location boxes to the AlphaPose network model in thread 2, which processes the fruit keypoints data. Thread 3 verifies the completeness of keypoint detection in the corresponding regions based on the target location and keypoint data from threads 1 and 2. It then outputs the keypoint locations, constructs the fruit picking region, and calculates the fruit pose vector.

### 2.5 Model training configuration

The hardware configuration used for model training in this study consisted of an Intel(R) Xeon(R) Gold 5218 CPU @ 2.30GHz processor and a Quadro RTX 5000 graphics card with 16GB of video memory. The operating system was Ubuntu 18.04, configured with CUDA 10.2 and CUDNN 8.6.0. Model training was performed within the Anaconda virtual environment installed on the system, with dependencies such as PyTorch 1.8.1 and mmcv-full 1.7.0 installed within the environment. The programming language used was Python 3.7. For training, the number of epochs was set to 300, the batch size to 32, the learning rate to 0.005, the optimizer to AdamW, and the number of workers to 4.

### 2.6 Indicators for model evaluation

Model testing was performed under the operating system Windows 11 and an RTX 4060 graphics card. To verify the performance of the keypoint detection model, the COCO keypoints dataset was used to evaluate the keypoint average precision (KAP) and average recall (KAR) metrics, as shown in (Equations 5, 6).


(5) $$KAP_{@\alpha }=\frac{\sum_{p}\delta (OKS_{p}>\alpha )}{\sum_{p}1}$$

(6) $$KAR_{@\alpha }=\frac{\sum_{p}\delta (OKS_{p}>\alpha )}{\sum_{gtp}1}$$

Where OKS(Object Keypoint Similarity) represents the keypoint recognition degree, *α* is the detection threshold for pineapple keypoints OKS. When the condition *δ*(OKS*_p_* >*α*) is satisfied, the value is set to 1; otherwise, it is set to 0. This means that the detected pineapple keypoints are considered positive if their score exceeds the threshold, and negative otherwise. Here, *p* represents the number of detected keypoints, and *gtp* is the number of the ground truth labeled keypoints. The OKS is shown as (Equation 7).


(7) $$OKS=\frac{\sum_{i}exp\left(-\frac{d_{i}}{2d^{2}K_{i}^{2}}\right)\delta (v_{i}>\alpha )}{\sum_{i}\delta (v_{i}>\alpha )}$$

Where *i* corresponds to the current number of the pineapple target keypoints group, *d_i_* is the Euclidean distance between the predicted pineapple keypoints and the ground truth labeled keypoints, *s* is the square root of the predicted pineapple target area, *K_i_* is the standard deviation constant for each type of pineapple keypoint, and *v_i_* indicates the visibility of the pineapple keypoints. Here, 0 means that the keypoints are not present in the real box, 1 means the keypoints are present but not visible, and 2 means the keypoints are visible.

## 3 Results and analysis

### 3.1 Comparison of different target detection models

In this study, the same dataset was used to train five YOLO series target detection models. The models were trained for 200 epochs, and the weight file with the best performance was selected as the final weight for the model. The performance comparison of each model is shown in **Table 1**, with Precision (P), Recall (R), and Average Precision (AP) of the YOLO series models used as the evaluation metrics (Khanam and Hussain, 2024).

**Table 1:** Performance comparison of different YOLO detection models.

Model	P	R	AP_0.5_	AP_0.5:0.95_	Speed/fps
YOLOv5n	97.1	98.6	99.3	89.1	102.0
YOLOv6n	98.0	**99.2**	**99.4**	**91.3**	**116.3**
YOLOv8n	**98.9**	98.7	**99.4**	90.7	108.7
YOLOv10n	97.0	98.7	**99.4**	90.6	84.0
YOLOv11n	98.9	97.8	**99.4**	90.1	86.2

Bold text indicates optimal values.

From the comparison results of different models, it can be observed that in terms of detection accuracy, the AP_0.5_ for YOLOv6n, 8n, 10n, and 11n models is all 0.994, with minimal difference between the five models. The AP_0.5:0.95_ for YOLOv6n is improved by 0.012 and 0.022 compared to YOLOv11n and YOLOv5n, respectively, and outperforms both YOLOv8n and YOLOv10n. Additionally, the recall of the YOLOv6n model is slightly better than the other four models, although its accuracy is 0.009 lower than that of YOLOv8n and YOLOv11n. In terms of detection speed, the YOLOv6n model demonstrates the best average detection speed of 116.3 fps, which is 14.3 fps, 7.3 fps, 32.3 fps, and 30.1 fps faster than the YOLOv5n, 8n, 10n, and 11n models, respectively.

Considering the comprehensive performance of the models, YOLOv6n demonstrates the best overall performance, with robust detection accuracy and speed. Therefore, this study uses the YOLOv6n model as the pineapple fruit target detection model.

### 3.2 Ablation test results

In order to evaluate the effectiveness of the improved model LTHRNet for pineapple fruit keypoint detection in this study, various enhancements were made to LiteHRNet using the LKA_stem(L) module, the D-Mixer(D) module, and the MS-FFN(M) module to construct five different STPEs, respectively. These models were evaluated using KAP and KAR values from the test set. The results of the performance comparison are shown in **Table 2**. The results revealed that the improved model LTHRNet detects pineapple fruit keypoints with KAP_0.5_ and KAR_0.5_ of 93.5% and 95.1% for the test set, which is 6.6%, 5.8%, 1.4%, and 6.6% higher than the other models using L, D, D-M, and L-D, with KAP_0.5_ and KAR_0.5_ boosted by 4.5%, 4.0%, 0.6%, and 3.8%, respectively.The results demonstrate that the introduction of the M module after the D module leads to a significant improvement in KAP_0.5:0.95_ and KAR_0.5:0.95_ for detecting pineapple fruit keypoints. This suggests that the model gains a richer feature representation capability after the M module integrates feature information from different scales. Furthermore, the model shows some improvement in each detection index after incorporating the L module. The final detection accuracy using the L-D-M model is optimal.

**Table 2:** Ablation test.

Module			KAP_0.5:0.95_	KAP_0.5_	KAR_0.5:0.95_	KAR_0.5_
L	D	M				
✓			73.8	86.9	82.2	90.6
	✓		75.8	87.7	83.1	91.1
	✓	✓	81.7	92.1	87.0	94.5
✓	✓		74.9	86.9	81.9	91.3
✓	✓	✓	**81.9**	**93.5**	**87.5**	**95.1**

**Figure 10:**
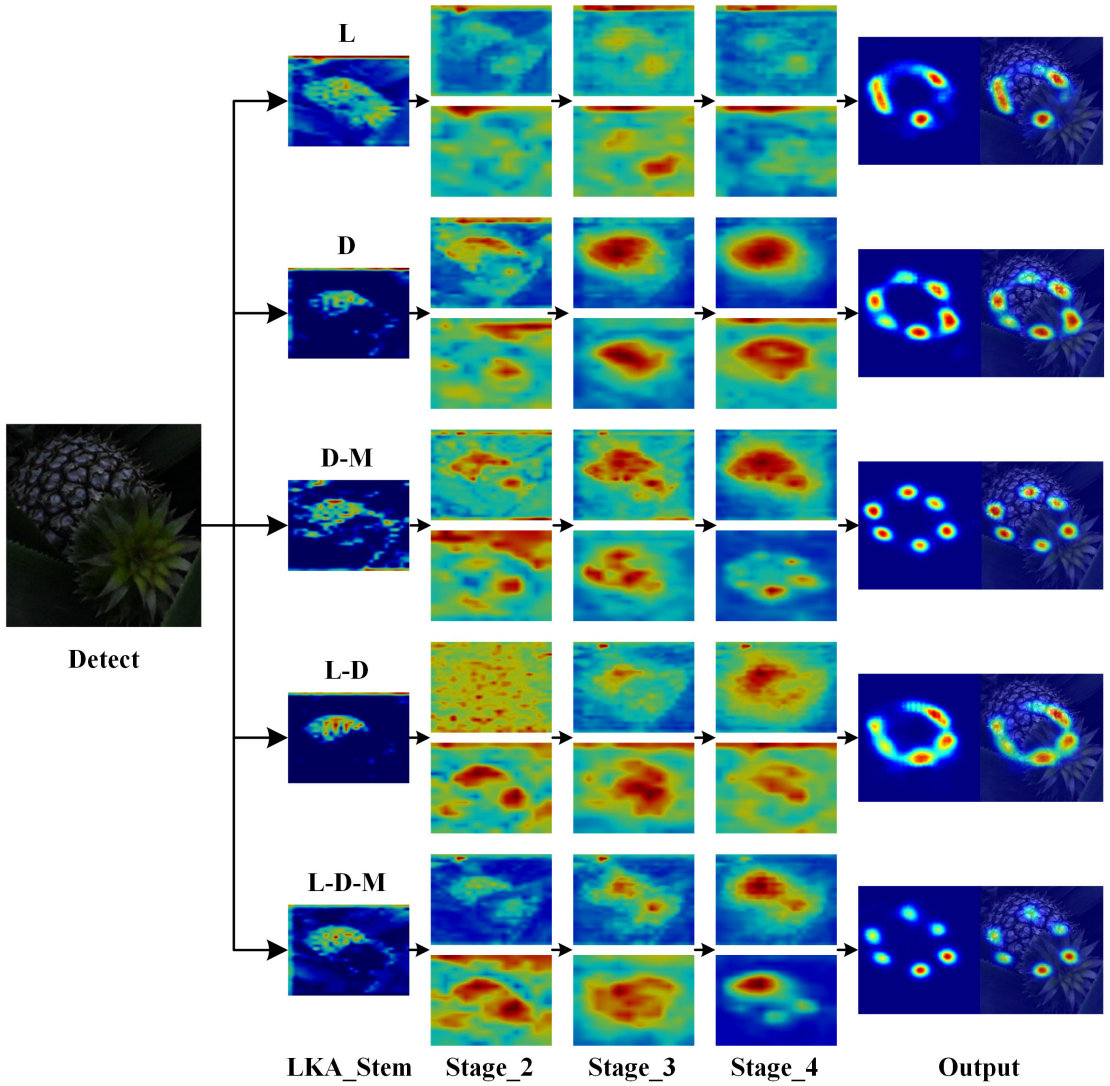
Visualization of the feature extraction process for each model of the ablation test.

The image feature extraction process for each model in the ablation experiment was visualized, with the final output feature maps of each stage and branch shown in **Figure 10**. The large kernel expansion convolution enlarges the receptive field, and a comparison of the LKA_Stem features across models reveals that using the LKA_Stem module enhances focus on the feature region and improves localization accuracy. This module increases the model’s sensitivity to internal features of the pineapple fruit, allowing it to capture subtle variations in fruit contour and texture more effectively while suppressing background noise.When comparing the *Stage* components of each model, it is evident that the use of the D-Mixer module concentrates attention on the fruit region. In the D-Mixer module, IDConv dynamically adjusts the convolution kernel based on input features, enabling it to effectively handle the variability in fruit morphology and reduce feature bias introduced by fixed convolution kernels. OSRA helps preserve boundary information of the fruit region and ensures accurate extraction of fruit features.From models using D and L-D configurations, some feature sticking is observed in the output features. However, in models using D-M and L-D-M, this feature stickiness is eliminated after introducing the M module. This module enhances attention to both local keypoint details and overall morphology via multi-scale convolution kernels, avoiding feature omissions that may occur with a single scale. It reinforces the contour information of the fruit, balances global and local features, and mitigates feature sticking.

### 3.3 Comparison of model test results

In order to evaluate the detection performance of the LTHRNet model for pineapple fruit keypoints in this study, a comparative test was established under different backbone network STPE to assess the prediction performance of LTHRNet against four other network models for pineapple fruit keypoint detection. The STPE of five different models were tested, and the performance indicators of each model are shown in **Table 3**. The results revealed that the LTHRNet model in this study improved KAP_0.5_ by 5.0%, 4.2%, 5.3%, and 11.9%, respectively, compared to four models: Fastpose, Simplepose, LiteHRNet, and TransXNet-T. KAR_0.5_ improved by 3.1%, 2.6%, 2.6%, and 7.0%, respectively. The detection speed was 21.1 fps, which is 5.2 fps and 7.7 fps faster than LiteHRNet and TransXNet-T, respectively. Although it was 10.2 fps and 14.0 fps slower than Fastpose and Simplepose, KAP_0.5:0.95_ improved by 4.6% and 4.9%, and KAR0.5:0.95 improved by 3.3% and 3.3%. Additionally, the number of model parameters was 22.4% that of Fastpose and 26.8% that of Simplepose. These results demonstrate that LTHRNet has an advantage in detection accuracy over other models. Therefore, the LTHRNet model proposed in this study is superior to other detection models in terms of comprehensive performance, with high real-time performance, and can provide accurate data for pineapple fruit pose localization.

**Table 3:** Performance comparison of different STPE models.

STPE	KAP_0.5:0.95_	KAP_0.5_	KAR_0.5:0.95_	KAR_0.5_	Params (M)	Speed/fps
Fastpose	77.3	88.5	84.2	92.0	40.6	31.3
Simplepose	77.0	89.3	84.2	92.5	34.0	**35.1**
LiteHRNet	75.6	88.2	84.1	92.5	**1.1**	15.9
TransXNet-T	62.4	81.6	73.4	88.1	12.4	13.4
LTHRNet	**81.9**	**93.5**	**87.5**	**95.1**	9.1	21.1

**Figure 11:**
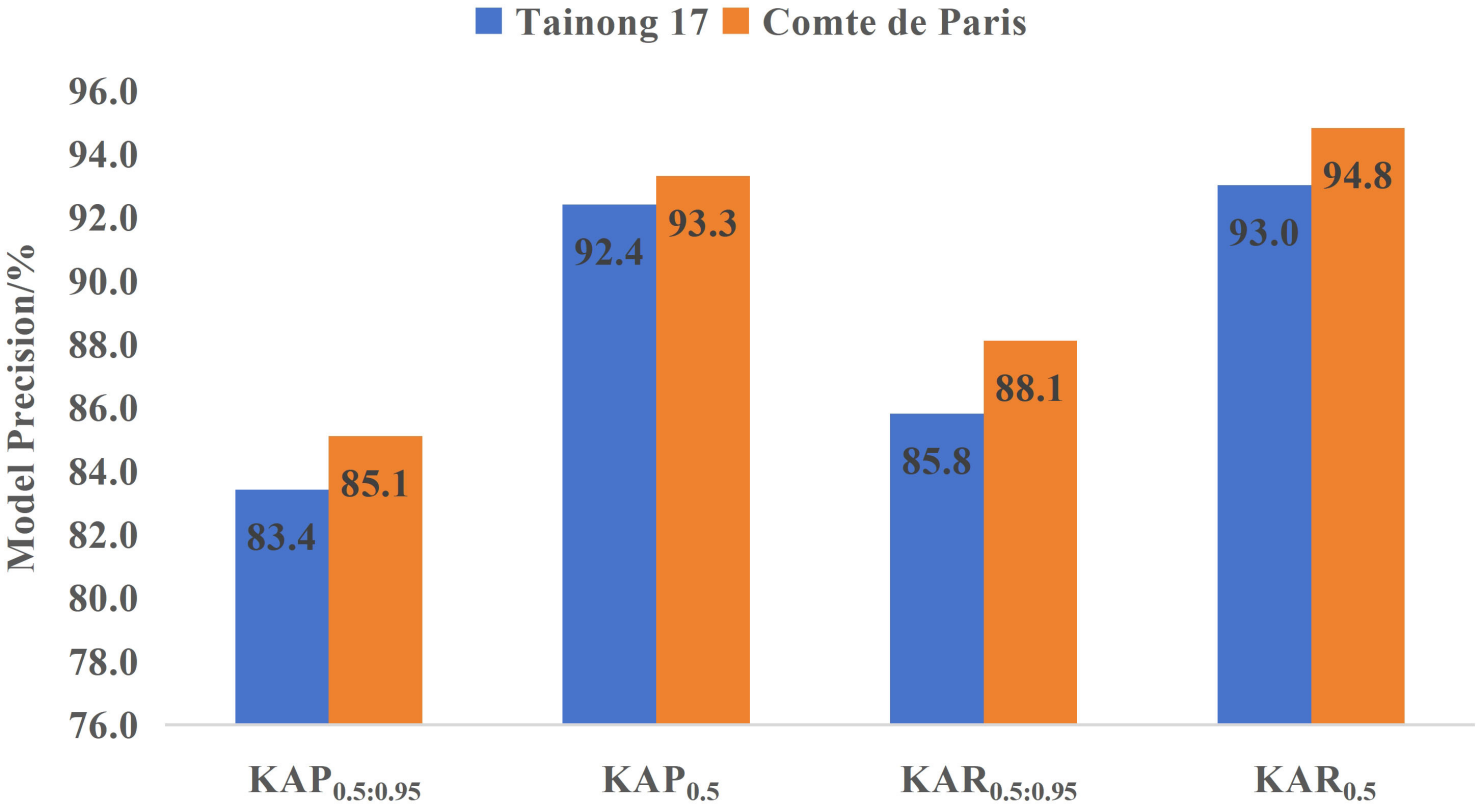
LTHRNet test results for different varieties.

Different pineapple varieties exhibit significant differences in characteristics such as individual fruit mass, core diameter, eye depth, and both longitudinal and transverse diameters (Zhang et al., 2022). Despite these variations, the model primarily relies on external visual features to localize keypoints. Notably, different pineapple varieties share strong visual commonalities, including their overall cylindrical shape and the surface texture characterized by a scale-like eye arrangement. As a result, the spatial distribution and relative positions of keypoints remain highly consistent across varieties. To verify the generalization ability of the LTHRNet model, external validation was conducted using 150 images each of Tainong 17 and ‘Comte de Paris’ pineapples. As shown in **Figure 11**, the KAP_0.5_ and KAR_0.5_ of Tainong 17 reached 92.4% and 93.0%, respectively, while those of ‘Comte de Paris’ reached 93.3% and 94.8%. Although the detection metrics for the ‘Comte de Paris’ variety were slightly higher, the overall differences in detection indices between the two pineapple varieties remained minimal. These results indicate that even with differences in longitudinal and transverse diameters, the projection relationships of keypoints in 2D images remain stable, enabling the model to maintain high detection accuracy across pineapple varieties. Therefore, the model demonstrates strong generalization capability.

### 3.4 Comparison of model detection effects

**Figure 12:**
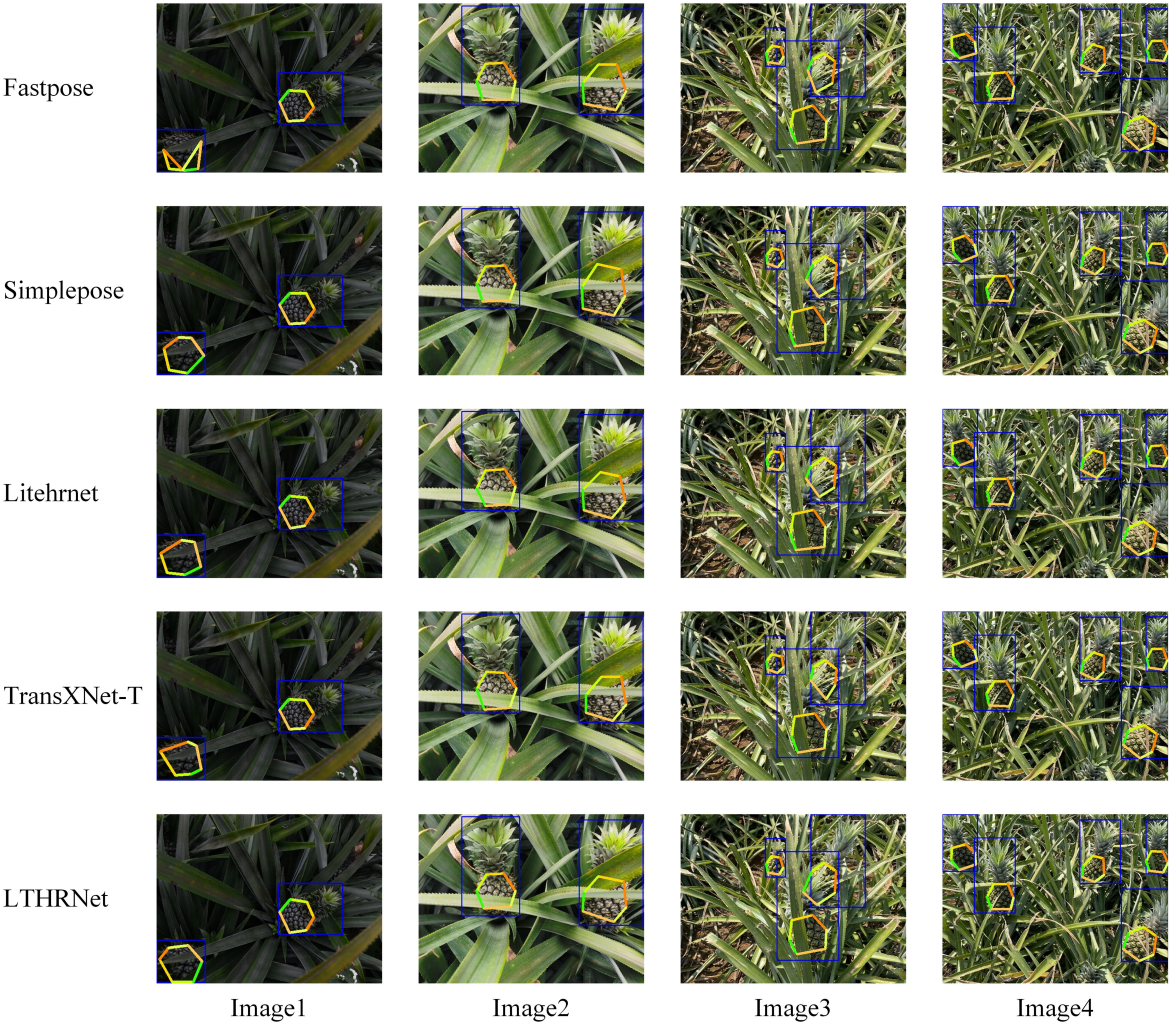
Detection results of different STPE models. (The blue rectangles represent the target detection results; the joint points of each differently colored line segment indicate the model-detected keypoints).

To verify the effectiveness of LTHRNet and the other four models in detecting the keypoints of pineapple fruits, the models were tested on pineapple fruit images under different lighting conditions. Some of the detection results, with the OKS threshold set to 0.5, are shown in **Figure 12**. The results reveal that Fastpose exhibits keypoint detection errors, while Simplepose, LiteHRNet, and TransXNet-T show large deviations and inaccurate keypoint locations, as illustrated in Image 1 on the lower left of the figure. Since the pineapple leaf may obscure the main part of the fruit, the other models experience issues with incorrect keypoint detection and significant location deviations, as seen in Image 2 and Image 3 in the figure. The proposed LTHRNet model performs well in addressing the issue of poor keypoint detection under occlusion. In conditions of low light intensity, detection difficulty increases, while in strong light intensity, the model more easily learns the features of the detected object, leading to accurate recognition of most pineapple fruit keypoints. Specifically, the LTHRNet model proposed in this study shows better detection results compared to the others. Simplepose produces similar detection results as LiteHRNet, while TransXNet-T has the poorest detection performance. These results demonstrate that LTHRNet can accurately recognize pineapple fruit keypoints under both complex backgrounds and varying lighting conditions.

Therefore, as shown in the keypoint detection results in **Figure 12**, the model in this study can accurately detect the keypoints at each location of the pineapple fruit. Even in occlusion scenarios, the corresponding keypoints are well detected, with more accurate localization compared to the other models, demonstrating better robustness in its localization.

### 3.5 Visualization of feature extraction

**Figure 13:**
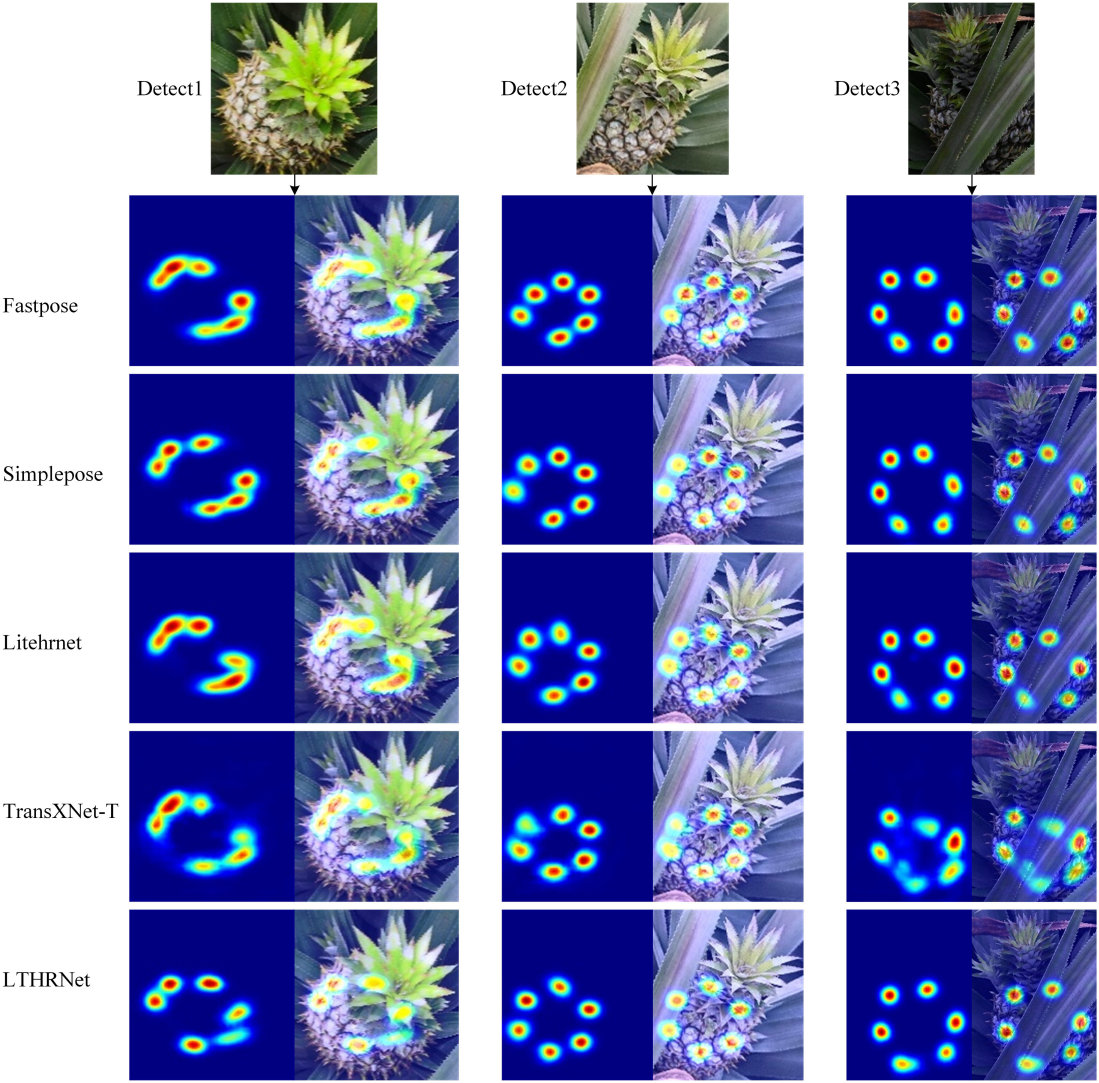
Different STPE model output feature visualization results.

In order to evaluate the feature extraction capability of the models in this study, LTHRNet was used to visualize the feature extraction process alongside the other four models. The visualized feature maps represent the final output feature maps of the models, and the results are shown in **Figure 13**. The results reveal that in the Fastpose, Simplepose, LiteHRNet, and TransXNet-T models, keypoints in Detect1 show redundant features that stick together. This feature sticking can easily cause the detected keypoint positions to be offset. In contrast, the output features of LTHRNet exhibit a lower degree of feature sticking, and the differentiation between each keypoint is more distinct compared to the other models.

In Detect1, the true positions of the keypoints in the image are close to each other, which leads to feature sticking in the model output. This issue is mainly caused by the small angle between the image and the pineapple crown bud. From the feature map of Detect1, it can be observed that LTHRNet performs better in extracting image features when the angle between the image and the pineapple crown bud is small. LTHRNet effectively filters redundant features and reduces feature sticking in adjacent keypoints.

In Detect2 and Detect3, some keypoints are blocked, and the final output features of TransXNet-T show the poorest feature localization accuracy. In contrast, the output features of the remaining four models exhibit some differences in feature attention location, with more noticeable variation in some feature areas. For the unobscured keypoints, each model demonstrates a strong ability to capture the corresponding features. In contrast, for the occluded keypoints, Fastpose and LTHRNet exhibit the strongest capability to capture keypoint features and the highest level of attention to feature areas. However, the feature area of Fastpose deviates more significantly from the actual location, whereas LTHRNet demonstrates greater accuracy in feature area localization.

In summary, the visualization results in **Figure 13** further confirm the high accuracy and stability of LTHRNet in feature extraction, indicating that LTHRNet has strong adaptability in handling challenging scenes.

### 3.6 Pose detection results

In this study, the real pineapple fruit pose orientation obtained from manual annotation data is compared with the orientation results estimated using the keypoints detection algorithm to evaluate the algorithm’s effectiveness in pineapple fruit pose detection. The orientation angle between the real fruit target and the detected target serves as the validation index, and its calculation is detailed in Equation 8, Equation 9.


(8) $$\vec{FP}={\vec{MP}}_{\left(p5,p6\right)}-{\vec{MP}}_{\left(p1,p2\right)}$$

(9) $$AOA=\frac{\sum_{i}∠\left({\vec{FP}}_{gi},{\vec{FP}}_{di}\right)}{N}$$

Here, **MP** represents the vector of the midpoints of the two keypoints, **FP** denotes the pineapple fruit facing vector, *N* is the total number of fruits detected in the test set, **FP**_g_ *_i_* and **FP**_d_ *_i_* are the facing vectors of the *i* true and detected targets, respectively, and AOA stands for the Average Offset Angle.

**Figure 14:**
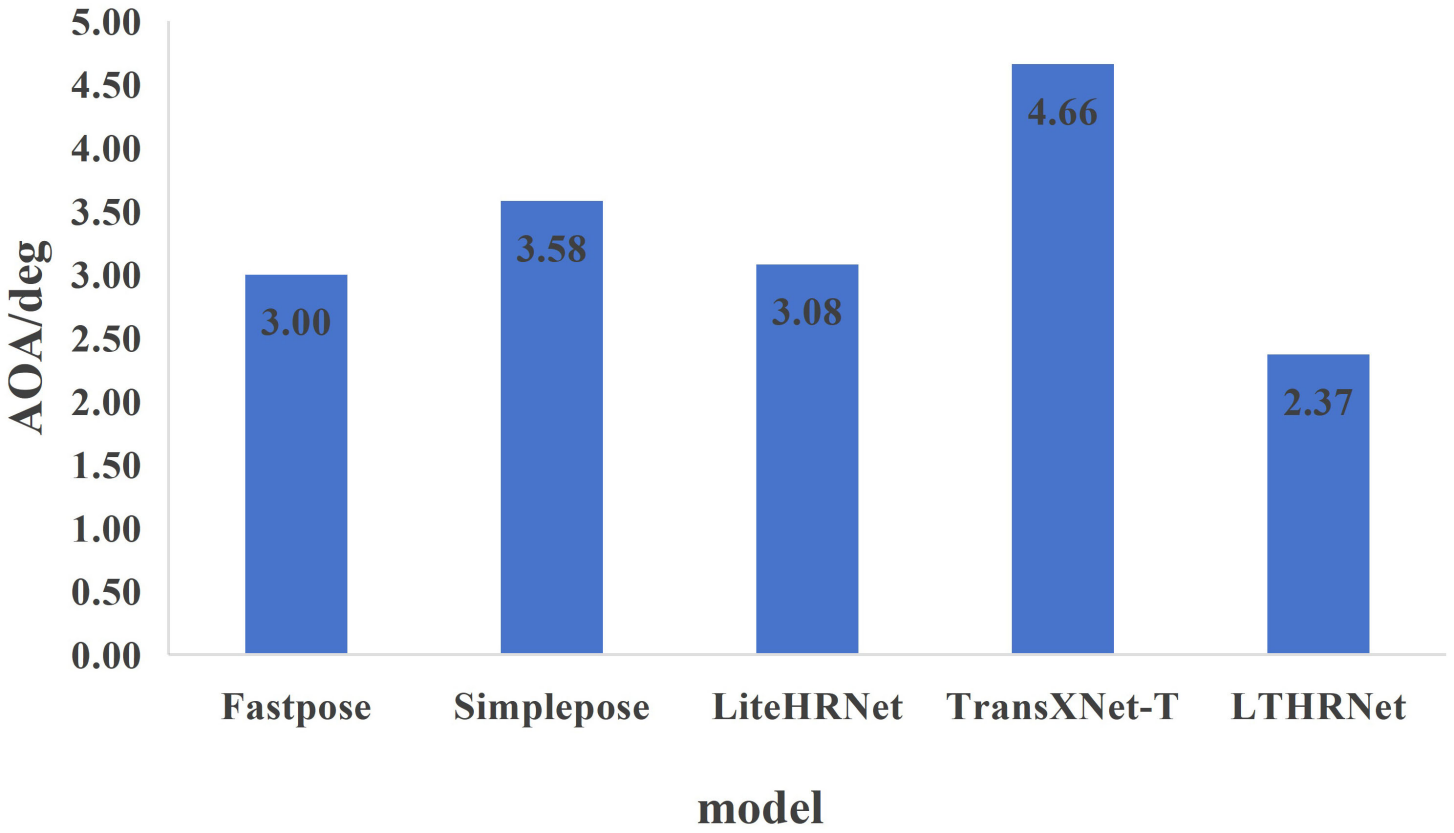
Model pineapple fruit pose prediction AOA.

The tested AOA values for pineapple fruit pose prediction across all models are presented in **Figure 14**. The results reveal that the LTHRNet model achieves the lowest AOA of 2.37°, while the TransXNet-T model exhibits the highest AOA of 4.66°. These results demonstrate that LTHRNet achieves the highest accuracy in pineapple fruit pose prediction, with the smallest offset between predicted and real attitudes, thereby providing reliable pose data.

**Figure 15:**
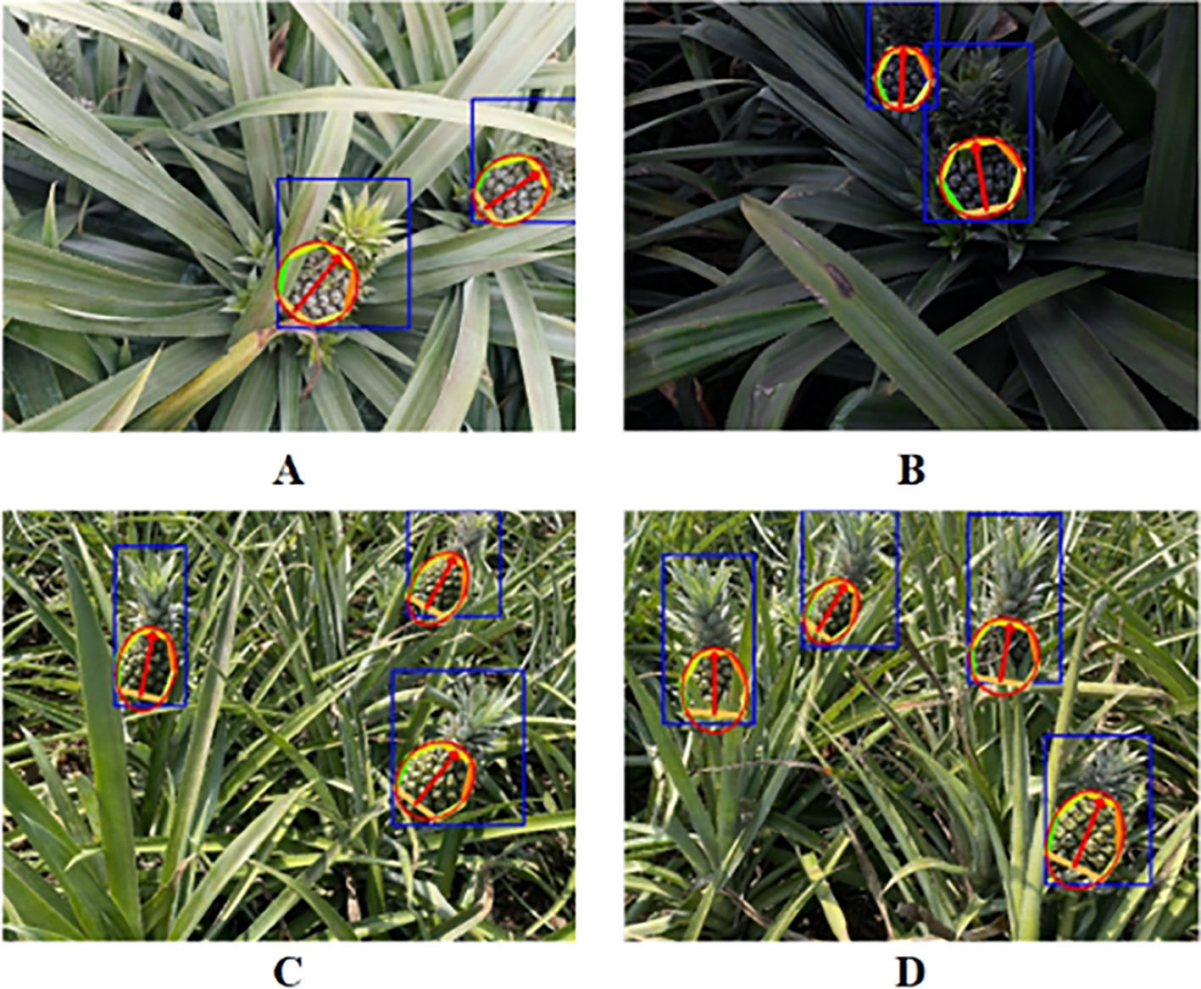
LTHRNet pineapple fruit pose detection results. **(A)** Cloudy. **(B)** Dusk. **(C)** Sunny. **(D)** Sunny with shadows.

**Figure 15** illustrates the results of LTHRNet pineapple fruit pose detection, demonstrating its ability to accurately recognize keypoints and predict fruit orientation under complex backgrounds, varying lighting conditions, and partial keypoint occlusion. Furthermore, the elliptical region fitted to the keypoints data more comprehensively captures the main part of the fruit, fulfilling the requirements for pineapple fruit picking region localization and pose prediction.

## 4 Discussion

In this study, a novel pineapple fruit keypoint detection model, LTHRNet, is proposed based on the improved LiteHRNet network structure. The model improvements include the introduction of the L module, the D module, and the M module, among others. The effect of each improved module on the model’s performance is evaluated through an ablation experimental system, which further demonstrates the effectiveness of the proposed method. The L module enhances the model’s feature extraction capability by combining large kernel convolutional decomposition with depthwise separable convolution. The D module boosts the model’s ability to capture both global and local features by integrating the advantages of CNN and Transformers. The M module further improves the model’s adaptability to complex features through multi-scale feature fusion. These improvements collectively lead to a significant increase in detection accuracy under various lighting and occlusion conditions.

The results of the ablation experiments show that LTHRNet outperforms other improved models in both KAP and KAR metrics, indicating that the multi-scale feature fusion mechanism of the M module plays a critical role in enhancing model performance. According to the experimental results, LTHRNet performs exceptionally well in pineapple keypoint detection under diverse lighting conditions and complex backgrounds. Furthermore, the feature extraction visualization results further validate that LTHRNet’s feature extraction ability is superior to that of other models, especially when the angle between the pineapple crown bud and the fruit body is small. This suggests that LTHRNet exhibits strong adaptability and stability when handling complex scenes. In terms of pose estimation, the AOA of LTHRNet is only 2.37°, which is significantly lower than that of other models.

LTHRNet, as a high-precision keypoint detection model, can provide core information on fruit pose, grasping regions, and target location, offering crucial data support for precise robotic arm operations. In the complete harvesting process, it is essential not only to locate the fruit but also to assess its ripeness. Moreover, due to the dense growth of pineapple leaves and significant leaf occlusion, the system must meet higher requirements for path planning. Based on the picking and grasping method, the required gripping force varies accordingly, imposing additional constraints on the harvesting strategy.

## 5 Conclusion

In this study, the proposed pineapple keypoint detection model, LTHRNet, based on the improved LiteHRNet network, aims to address the challenge of fruit pose detection in pineapple fruits with complex backgrounds and lighting conditions. By enhancing LiteHRNet and introducing the L, D, and M modules, the keypoint detection accuracy of the model and the accuracy of pose prediction are significantly improved. The main conclusions of the study are as follows:

1. LTHRNet performs exceptionally well in the pineapple fruit keypoint detection task, with the model’s detection accuracy and robustness significantly surpassing that of the other compared models under complex backgrounds, varying lighting conditions, and occlusion. The experimental results show that LTHRNet achieves a substantial improvement in both KAP_0.5_ and KAR_0.5_, reaching 93.5% and 95.1%, respectively.
2. LTHRNet demonstrates superior feature extraction capability and higher feature differentiation in feature extraction visualization experiments. It is able to effectively filter redundant features and reduce feature sticking when dealing with adjacent keypoints’ feature sticking and occlusion situations.
3. LTHRNet excels in pineapple fruit pose estimation, accurately estimating the pineapple fruit’s pose orientation with a minimal offset of just 2.37°from the true pose. By accurately detecting the keypoints of pineapple fruits and estimating their poses, the model provides reliable data support for automated picking systems.

In future work, we will focus on fruit ripeness detection, incorporate reinforcement learning to optimize path planning, and dynamically adjust the gripping strategy based on keypoint fitting and fruit morphological parameters. These improvements will be integrated into mobile picking robots to achieve intelligent pineapple harvesting.

## Data Availability

The original contributions presented in the study are included in the article/supplementary material. Further inquiries can be directed to the corresponding author/s.
